# Lipid-Based Nanoparticles as a Pivotal Delivery Approach in Triple Negative Breast Cancer (TNBC) Therapy

**DOI:** 10.3390/ijms231710068

**Published:** 2022-09-03

**Authors:** Aiswarya Chaudhuri, Dulla Naveen Kumar, Rasheed A. Shaik, Basma G. Eid, Ashraf B. Abdel-Naim, Shadab Md, Aftab Ahmad, Ashish Kumar Agrawal

**Affiliations:** 1 Department of Pharmaceutical Engineering and Technology, Indian Institute of Technology (BHU), Varanasi 221005, India; 2 Department of Pharmacology and Toxicology, Faculty of Pharmacy, King Abdulaziz University, Jeddah 21589, Saudi Arabia; 3 Department of Pharmaceutics, Faculty of Pharmacy, King Abdulaziz University, Jeddah 21589, Saudi Arabia; 4 Health Information Technology Department, Faculty of Applied Studies, King Abdulaziz University, Jeddah 21589, Saudi Arabia

**Keywords:** liposomes, nanoemulsion, solid lipid nanoparticles, nanostructured lipid carriers, lipid–polymer hybrid nanoparticles, triple-negative breast cancer, targeted therapy

## Abstract

Triple-negative breast cancer is considered the most aggressive type of breast cancer among women and the lack of expressed receptors has made treatment options substantially limited. Recently, various types of nanoparticles have emerged as a therapeutic option against TNBC, to elevate the therapeutic efficacy of the existing chemotherapeutics. Among the various nanoparticles, lipid-based nanoparticles (LNPs) viz. liposomes, nanoemulsions, solid lipid nanoparticles, nanostructured lipid nanocarriers, and lipid–polymer hybrid nanoparticles are developed for cancer treatment which is well confirmed and documented. LNPs include various therapeutic advantages as compared to conventional therapy and other nanoparticles, including increased loading capacity, enhanced temporal and thermal stability, decreased therapeutic dose and associated toxicity, and limited drug resistance. In addition to these, LNPs overcome physiological barriers which provide increased accumulation of therapeutics at the target site. Extensive efforts by the scientific community could make some of the liposomal formulations the clinical reality; however, the relatively high cost, problems in scaling up the formulations, and delivery in a more targetable fashion are some of the major issues that need to be addressed. In the present review, we have compiled the state of the art about different types of LNPs with the latest advances reported for the treatment of TNBC in recent years, along with their clinical status and toxicity in detail.

## 1. Introduction 

The American Cancer Society and National Cancer Institute reported approximately 276,480 new cases of breast cancer in the year 2020, out of which approximately 42,170 women died. Further, it was reported that in comparison to every 87 new cases per 100,000 women of HR+/HER2− type breast cancer, TNBC accounts for 13 new cases per 100,000 women. In addition, the American Cancer Society also reported that TNBC exhibited the worst 5-year relative survival rate (≈76.7%), compared to other types of breast cancer (≈90%). Moreover, as TNBC has a reputation for showing metastasis to the brain, lungs, and bones, its stage plays an important role in determining survival outcomes, which states that if the cancer is localized, then survival rate is ≈80.2%, but it eventually drops to ≈11.5% if the cancer is metastasized [1]. TNBC is characterized by alteration in signaling pathways, overexpression of oncogenes, and absence of hormonal (ER, PR, HER-2) expressions, which restrict the treatment to surgery, chemotherapy, radiation, and immunotherapy [2,3]. However, when TNBC gets distant, chemotherapy is considered the most widely employed treatment option with increased survival rates [4].

Although chemotherapy is a widely employed anticancer treatment strategy, it does display certain significant toxicities such as immunosuppression, bone marrow suppression, mucositis, alopecia, gastrointestinal discomfort, ventricular dysfunction, anemia, fatigue, etc., mainly due to its non-selectivity and non-specific cytotoxic effects over healthy cells of bone marrow, gastrointestinal epithelial, and hair follicles. Moreover, chemotherapy also shows multi-drug resistance (MDR) that decreases the anticancer activity of the chemotherapeutics which hinders their efficacy at the cancer site [5]. In addition to this, the drugs used in chemotherapy also display certain limited biopharmaceutical attributes such as large size, metabolic instability, poor aqueous solubility, and susceptibility to p-glycoproteins that hinder their delivery to the cancer site [6,7]. Such findings necessitate the development of a smart therapeutic strategy that will improve the intrinsic biopharmaceutical attributes of the chemotherapeutics as well as facilitate targeted delivery of chemotherapeutics to the cancer site preventing the proliferation and metastasis and enhancing their therapeutic efficacy [8].

This situation led to the emergence of cancer nanotechnology that overcomes the drawbacks of conventional drug delivery systems starting from small-scale barricades such as intracellular trafficking and site-specific targeting to large-scale barriers such as biodistribution [9,10]. Further, to initiate various studies and carry them to clinical translation, in the year 2000, the National Nanotechnology Initiative (NNI) was launched by the US National Science and Technology Council (NSTC) for supporting and improving nanotechnology research. For such initiation, nanoparticles (NPs) have gained much attention in the drug delivery system against various diseases such as cancer [11], diabetes [12,13,14,15], and bacterial infection [16,17,18]. Recently, nanoparticles were employed for the delivery of vaccines [19,20], proteins [21], nucleic acid [22,23], etc. Such application has enhanced the concept of nanotechnology in the field of medical science. Among various nanoparticles (NPs) for cancer therapy, lipid-based NPs (LNPs) are recognized as the most widely approved class of nanoparticles by FDA due to various advantages such as simplicity in their fabrication, ability to self-assemble in aqueous media, offering enhanced bioavailability, being biocompatible, ability to load both hydrophilic and hydrophobic drugs, increased loading capacity, ability to modulate their surface characteristics, etc. [24].

In this review, we discuss the various LNPs employed for the treatment of TNBC such as liposomes, nanoemulsions (NEs), solid lipid nanoparticles (SLNs), nanostructured lipid carriers (NLCs), lipid–polymer hybrid nanoparticles (LPH-NPs), and exosomes (Exo) with emphasis on properties of various lipids employed in the fabrication of LNPs, that display their characteristic mechanism of loading of drugs and delivering them to the cancer site. We also mention the current challenges and future perspectives of LNPs in the effective treatment of TNBC.

## 2. Lipid-Based Nanoparticles: A Versatile Drug Delivery System

Rapid advances in lipid-based nanoparticles show an immense impact on cancer treatment and management. Looking into the structural makeup of LNPs, we found that LNPs are composed of lipids that are both biodegradable and biocompatible such as phospholipids, cholesterol, and triglycerides. The inclusion of such less toxic substances, as well as limited use of organic solvents, makes LNPs a safe drug delivery system against TNBC as compared to polymeric and inorganic NPs [4,8]. According to the general prototype, LNPs consist of API, lipids that are designed to sequester, deliver, and promote functionality. Further, to maintain the stability of the dispersions formed by the LNPs, in environmental stresses, surfactants or a combination of surfactants are used, depending on their HLB values [25]. Further, to safeguard the LNPs from the reticuloendothelial system (RES) and to render biological stability, LNPs are often coated with a stealth or biocompatible polymeric layer such as polyethylene glycol (PEG) [6,26,27]. Finally, for possessing an enhanced targeting property, the LNPs may bind with a targeting moiety such as a biological receptor-specific ligand. Such orientation of LNPs and their small size help the LNPs to accumulate frequently on the tumor sites [6].

In cancer research, it was observed that approximately 40% of new chemical entities show poor aqueous solubility, which further limits their therapeutic efficiency. Thus, the utilization of hydrophobic LNPs as a vehicle either encapsulates or solubilizes the drug moiety that will further improve the stability of poorly aqueous soluble drugs in the surrounding aqueous media and will prevent the precipitation of the drugs both in vitro and in vivo. Hence, it could be inferred that the development of LNPs shows improved chemotherapeutic efficacy by increasing their solubility [28,29]. It was further revealed that the absorption of the poorly water-soluble chemotherapeutics in the LNPs is due to the increased solubilizing capacity of the lipids/oils. The other well-established strategies employed by LNPs in increasing the solubility of the poorly water-soluble chemotherapeutics, thereby increasing their bioavailability, include preservation of the chemotherapeutics within the lipidic matrix against chemical and enzymatic degradation, alteration in the permeability of the gastrointestinal membrane, and facilitation of the lymphatic drug transport [25], as described in Table 1.

**Figure 1:**
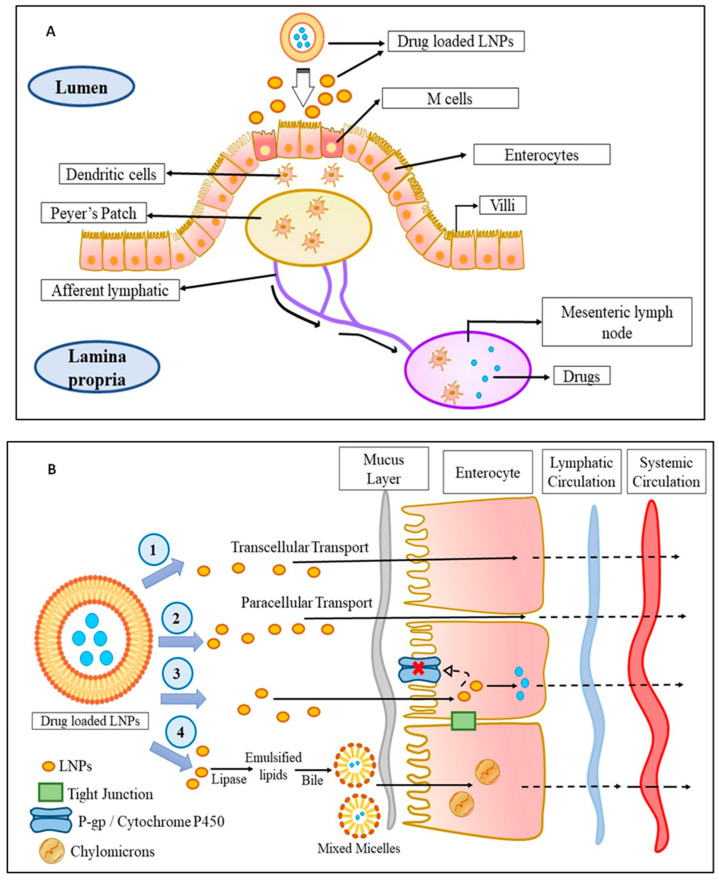
The mechanism of LNPs uptake into the lymphatic circulation: (**A**) Uptake of drug-loaded LNPs by Peyer’s patch into the lymphatic system: The drug-loaded LNPs are taken up by the M-cells of the enterocytes which are then taken up by the dendritic cells followed by Peyer’s patch from where the drug-loaded LNPs enter into the lymphatic system via afferent lymphatic. (**B**) Uptake of drug-loaded LNPs by an intestinal wall into the lymphatic system: The drug-loaded LNPs enter the lymphatic system through the intestinal wall in fours ways—(1) transcellular transport, (2) paracellular transport, (3) by inhibiting P-gp glycoprotein and cytochrome P450, or (4) by the production of chylomicrons. Abbreviations: M cell: membranous cell, LNPs: lipid-based nanoparticles; P-gp: P-glycoprotein.

Talking in terms of oral delivery, most chemotherapeutics are suffered from first-pass metabolism which limits their concentration in blood and subsequently at the cancer site, which restricts their therapeutic efficacy. In this context, it was found that drug delivery through the lymphatic system overturns the first-pass metabolism and improves their targeting to the cancer site. From various studies, it was observed that the triglycerides, cholesterol esters, and lipid-soluble vitamins are easily taken up by the lymphatic system and as the LNPs are mostly composed of triglycerides and arranged similarly to that of chylomicrons, the LNPs are easily taken up by the lymphatic system, thereby circumventing the first-pass metabolism, improving their targeting, and reducing toxicity. Physiologically, it was observed that the lymphatic uptake of LNPs occurs via three routes as shown in Figure 1. First is through the gaps occurred within the lymphatic capillaries, the second is through the Peyer’s patches (Figure 1A), which are either isolated or aggregated lymphoid follicles, and the third is through the intestinal wall which follows four different mechanisms, namely, transcellular absorption, paracellular absorption, inhibition of the activity of P-glycoprotein, and cytochrome P450 and generation of chylomicrons (Figure 1B) [32].

Like cellular uptake, the in vivo fate of the LNPs also plays an important role in the drug delivery system. Basically, the entrapped drug gets released into the physiological surrounding only after breaking of the lipidic matrix. Such phenomenon occurs via lipolysis wherever lipases are found in abundance, especially in the GIT or surface erosion, in case the lipids are insensitive to lipolysis. It was observed that the LNPs composed of aliphatic esters are rapidly degraded by lipases, especially in the small intestine, while the LNPs comprised of triglycerides were first broken down by lysosomal acid lipases into diglycerides, which was then broken down into monoglycerides, and finally into fatty acids in the GIT, followed by endocytosis. Then, the lipolysates form and along with the encapsulated drugs, both are transported to the epithelial surfaces in the form on vesicles or micelles for absorption. It was further observed that apart from GIT, the lipolysis also takes place within tissues and cells. In the process of erosion, the lipid matrix undergoes either hydrolysis or dissolution which eventually plays a role in complete degradation of lipid matrices based on fatty acids and are insensitive to lipolysis. It was observed that as the chain length of the fatty acid increases, the rate of erosion of lipid matrix declines, and drug release becomes slow and steady, while the lipids with medium chain length significantly increases the erosion rate of the lipid matrix, which thereby increases the drug release from the lipid matrix [33]. 

It was further observed that the membrane-like structure of LNPs provides flexibility in their particle sizes which enables them to stay in the systemic circulation for a longer period by bypassing the immune responses, resulting in improved passive accumulation of LNPs in the cancer site [34]. Moreover, the particle sizes of LNPs are generally greater than 10 nm in diameter, which restricts their elimination by the kidney but allows the elimination via capillaries situated at the leaky microvasculature. Since leaky microvasculature is a common characteristic of solid tumors such as TNBC, many potent chemotherapeutics have been encapsulated in LNPs to extract the advantage of the EPR effect, which further results in increased drug accumulation at the tumor site with reduced dose regimen and systemic side effects. In this context, the encapsulation of doxorubicin (DOX) within LNPs (Doxil^®^/Caelyx^®^) increased their tumor accumulation and reduced their distribution to the myocardium, thereby decreasing the doxorubicin-induced cardiotoxicity, as compared to conventional doxorubicin solution (Adriamycin^®^, Pfizer, Manhattan, NY, USA) [28].

It was observed that the challenging task in the preparation of LNPs is obtaining proper size and polydispersity of the LNPs and uniform loading of chemotherapeutics within the LNPs. It was observed that the desired shape and size was obtained by controlling the process parameters employed in the various types of preparation procedure of LNPs. For instance, high pressure homogenization is one of the methods employed for the preparation of LNPs. It was observed that during hot high-pressure homogenization, very narrow particle size distribution was obtained, while in cold high-pressure homogenization, a broad size distribution was obtained. It was inferred that the size distribution of LNPs in high-pressure homogenization depends upon the temperature and pressure provided, type of homogenizer employed, and number of homogenization cycles applied. Likewise, in solvent emulsification evaporation method, the particle size of LNPs were controlled by the type and concentration of lipids, and surfactant mixture within the organic phase. It was observed that the particle size ranges between 30 and 100 nm when the lipid concentration is employed up to 5% *w*/*v*, above which the particle size increases beyond 100 nm. In case of solvent emulsification diffusion method, the particle size obtained was below 100 nm with narrow particle size distribution. It was observed that particle size increases on usage of non-ionic surfactant, while it decreases on using ionic surfactant. However, it was suggested to use a combination of two or more surfactants for better control of the particle size. Lastly, in the ultrasonication method, the particle size is obtained in the range of 30–200 nm with broad particle size distribution. It was observed that the particle size can be controlled by varying the frequency, intensity, and time of ultrasonication [35].

For the loading of the chemotherapeutics within the LNPs, the active incorporation method can be used, i.e., loading of drugs after LNPs formation, or passive i.e., loading of drugs during LNPs formation [36]. The active method involves adsorption or absorption methods that are achieved by incubating the LNPs with concentrated drug solution [37]. The passive method involves the mechanical method, solvent dispersion method, and detergent removal method [36]. It was observed that the drug loading depends upon the solubility of the drugs within the lipid matrix, which is further associated with the composition of the lipid matrix, molecular weight of the drug, the interaction between the drug and lipids and the presence of end functional groups (i.e., ester or carboxyl) in either the drug or lipid matrix [37]. LNPs were also used for the loading of nucleic acid (siRNA, mRNA, and pDNA) proteins. It was observed that fabrication of nucleic acid loaded LNPs include detergent dialysis and ethanol loading technique. However, the rapid-mixing method and T-mixing method have gained more popularity as it assures >90% entrapment efficiency. In recent times, microfluidic mixing approaches were designed based on rapid-mixing approach which further promises to fabricate nucleic acid, and protein loaded LNPs in a more reproducible and scalable fashion. It was further observed that all the mentioned methods allow rapid mixing of lipid containing organic phase into aqueous phase comprised of nucleic acid, and proteins, and resulting in an enhanced entrapment efficiency [38].

**Figure 2:**
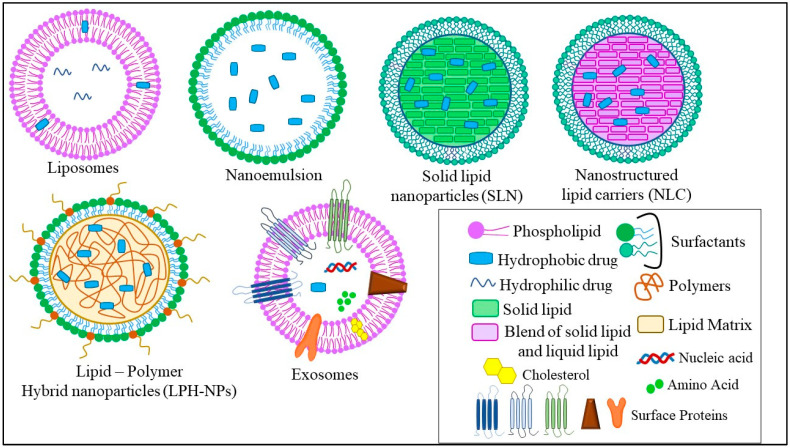
Different types of LNPs used for the treatment of TNBC.

The different types of LNPs developed for the treatment of TNBC are liposomes, nanoemulsions (NEs), solid lipid nanoparticles (SLNs), nanostructured lipid carriers (NLCs), lipid–polymer hybrid nanoparticles (LPH-NPs), and exosomes (Exo). Briefly, liposomes are microscopic phospholipid bilayer nanovesicles while NEs are colloidal nanosystems with lipophilic surfaces and a negative charge. SLNs are colloidal nanosuspensions, while NLCs are colloidal blends of solid and liquid lipids. LPH-NPs are colloidal blends of lipids and polymers (non-lipid substances) [4]. Exosomes (Exo) are biological nanosized vesicles composed of lipid bilayers with embedded surface proteins [39]. The distinct characteristics, advantages, and disadvantages of each type of LNPs are mentioned in Table 2 and described in Figure 2.

### 2.1. Liposomes

Liposomes are a vesicular-type drug delivery system obtained spontaneously by dispersing the lipids (phospholipids) in aqueous media. The liposomes were first discovered in 1963 by Alec Bangham [47]. In terms of lipid shell, phospholipids form the main element of liposomes [48]. It was found that phospholipids are also considered an important constituent of the biological membrane. The phospholipids are comprised of a polar head, which is composed of hydrophilic moieties, and a non-polar tail, which is comprised of hydrophobic moieties [49,50]. Depending on the presence or absence of charges or the type of charges, liposomes are classified as uncharged, positively charged, negatively charged, and amphiphilic (zwitterionic). The positively charged lipids used in the formation of liposomes are N-[1-(2,3-dioleyloxy)propyl]-N,N,N-triethylammonium (DOTMA), and 1,2-dioleoyl-3-trimethylammoniopropane (DOTAP), while the negatively charged lipids employed in liposomes are phosphatic acid, phosphatidylserine, phosphatidylglycerol, phosphatidylinositol, and dicetylphosphate). The various zwitterionic lipids used are phosphatidylethanolamine, phosphatidylcholine, etc. [51]. It was found that the administration of the charged lipids increases the interlamellar distance between the phospholipid bilayers, which provides enhanced drug entrapment efficiency and physical stability. Further, in a study, it was observed that the presence of DMPC (dimyristoylphosphatidylcholine, a derivative of phosphatidylcholine) induced apoptosis in various cancer cell lines such as breast cancer, lung cancer, etc. [47]. Apart from phospholipids, cholesterol also exists as one of the elements of liposomes and plays a vital role in maintaining and preserving the fluidity, permeability, and stability of the phospholipids both in vitro and in vivo. Further, it was observed that using derivatives of cholesterol such as 6-aminomannose-cholesterol or glycosylated cholesteryl bypasses the RES uptake and increases the targetability of the liposomes towards cancer cells [47]. It was further found that the liposome also improves the aqueous solubility of poorly water-soluble drugs [28].

Therefore, from the above reports, it was inferred that the stability of the phospholipid bilayer, the entrapment efficiency, and the drug loading of the liposomes as well as their tissue distribution and renal clearance ultimately depend on the composition of the lipid membranes and the content of the cholesterol.

Various poorly water-soluble drugs have been administered using liposomes as a delivery system such as indomethacin, amphotericin B, and azidothymidine, which have already reached the commercial market [28].

**Figure 3:**
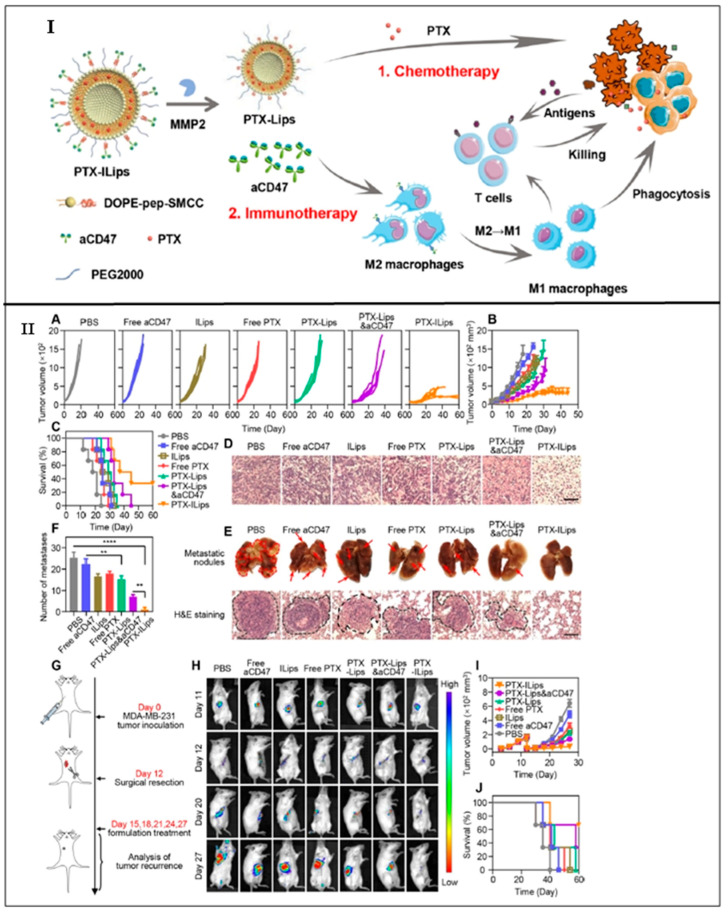
(**I**) An illustration displaying the construction of PTX-ILips and the release of PTX and aCD47 from the liposome for effective chemotherapy and immunotherapy respectively against TNBC. (**II**) Anticancer efficacy study and recurrence inhibition study in vivo: (**A**) Individual tumor growth curves in different groups. (**B**) Tumor growth kinetics of MDA-MB-231 tumors in mice treated with different formulations. (**C**) Survival rates of animals in various groups. (**D**) H&E staining of tumor slices collected from mice after the treatment of 21 days. Scale bar = 100 μm. (**E**) Photographs of lung metastatic nodules and histological assessment of lung metastatic nodules via H&E staining. Scale bar = 100 μm. (**F**) Numbers of lung metastatic nodules from each group. (**G**) Schematic illustration of the establishment of tumor recurrence model and therapy with different formulations. (**H**) Representative IVIS images of MDA-MB-231 tumor-bearing mice in each group. (**I**) Tumor volume growth curves after tumor implantation, subsequent surgery, and therapy. (**J**) Survival of mice in different treatment groups. Data are displayed as the mean ± SD. ** *p* < 0.01; **** *p* < 0.0001. Reprinted (adapted) with permission from [54]. Copyright (2021) American Chemical Society.

Guo et al., 2019 developed a dual complementary liposome (DCL) composed of lipids such as 1,2-dioleoyl-sn-glycero-3-phosphocholine (DOPC) and 1,2-distearoyl-sn-glycero-3-phosphoethanolamine-N-[carboxy (polyethylene glycol)-2000] (DSPE-PEG-COOH), encapsulating doxorubicin and further surface-functionalized with antibodies against intercellular adhesion molecule–1 (ICAM1) and epithelial growth factor receptor (EGFR) for effective treatment of TNBC. It was observed that DCLs showed an average particle size of 130 ± 30 nm with a zeta potential of −6 and −10 mV. Further, it was revealed that DCLs showed enhanced internalization to MDA-MB-231 cells and MDA-MB-436 cells (42.7% and 60.9% respectively), along with a significant reduction of proliferation in vitro (30–40%). Moreover, the cancer cells (MDA-MB-231 and MDA-MB-436) treated with DCLs showed a reduction in their cell count by 64% and 46%, respectively. DCLs displayed enhanced tumor targetability and antitumor efficacy with reduced lung metastasis, depicting that the DCLs could be served as an effective therapeutic nanoplatform against TNBC [52]. Yan et al., 2019 fabricated tLyp-1-peptide modified liposomes composed of DSPE-PEG2000. The modified liposomes were prepared to encapsulate the miRNA responsible for silencing the slug gene. It was found from the previous studies that the slug gene is responsible for activating the TGF-β1/Smad pathway, causing invasion and proliferation of TNBC cells. It was found that the modified miRNA liposomes showed a particle size of 120 nm and exhibited an enhanced cellular uptake by TNBC cells in vitro, targeting mitochondria. Moreover, the modified miRNA liposomes showed enhanced anticancer activity and silenced the expression of the slug gene. In addition to these, the modified miRNA liposomes showed increased internalization to TNBC cells (48.79 ± 0.42), as compared to free miRNA complexes (3.69 ± 0.08). In addition, modified miRNA liposomes showed increased inhibitory rates (64.33 ± 8.18%), as compared to free miRNA complexes [53]. Chen et al., 2021 prepared detachable immune liposomes (ILips) as an immunochemotherapeutic approach for delivering paclitaxel and anti-CD47 in the TNBC. The lipids used for the preparation of ILips include DOPE (dioleoylphosphatidylethanolamine). It was observed that the ILips facilitate the release of CD-47 in response to MMP2 eventually polarized the M2 phenotype to M1 macrophage enhancing phagocytosis of TNBC cells and activating the responses of the T cell immune system. Paclitaxel and CD-47 showed a synergistic anticancer effect along with reduced metastasis, compared to paclitaxel liposomes and free CD-47 (2.3- and 3.1-fold, respectively). Furthermore, a lower IC_50_ was observed in the case of ILips as compared to paclitaxel-liposomes and free paclitaxel (2.8- and 6.4-fold respectively), which indicated that the ILips showed significant inhibition of TNBC cell proliferation. In addition to these, ILips showed an increased expression of CD80 (1.5-fold) as compared to free CD-47, indicating that the binding of PTX and CD-47 led to the effective delivery of CD47 to cancer sites along with increased polarization of macrophages (Figure 3) [54]. Alawak et al., 2021 engineered thermoresponsive liposomes encapsulating doxorubicin for the effective treatment of TNBC. The engineered thermoresponsive liposomes were further surface-functionalized by linking with the MAB1031 antibody via covalent coupling (LipTS–GD–MAB). It was observed that the MAB1031 antibody was employed to target ADAM8, found to be overexpressed in TNBC patients. The lipids used for the fabrication of liposomes include DPPC, DSPC, cholesterol, and DSPE. The cellular toxicity study revealed that 80% of cells were found viable when the cells were treated with LipTS–GD–MAB. In addition to this, the LipTS–GD–MAB showed increased cellular internalization as compared to doxorubicin liposomes [55]. El-Senduny et al., 2021 prepared Azadiradione-loaded liposomes (AZD-lipo) for effective treatment of TNBC. It was observed that the AZD-lipo showed enhanced anti-cancer activity along with increased oral bioavailability as compared to free AZD. In addition, AZD-lipo showed less expression of proteins responsible for the proliferation of TNBC cells, and angiogenesis in TNBC cells such as cyclin D1, COX-2 (0.024 ± 0.005 at 50 μM), survivin, and VEGF-A (0.302 ± 0.01 at 25 μM) as compared to free AZD, where the concentration of COX-2 is 0.553 ± 0.015 at 50 μM, and that of VEGF-A is 0.801 ± 0.011 at 25 μM. In addition, the AZD-lipo showed decreased IC_50_ values (26.85 ± 3.48 μM), as compared to free AZD (44.88 ± 2.57 μM). Such observations indicated an increased bioavailability of AZD in the biological system from AZD-lipo. Hence, it could be inferred that liposomes provide an effective therapeutic strategy for increased delivery of low bioavailable drugs and effective treatment against TNBC [56].

### 2.2. Nanoemulsions (NEs)

Nanoemulsions (NEs) are an isotropic thermodynamically stable system composed of two immiscible liquids that are equilibrated into a monophase using surfactants or a mixture of surfactants and co-surfactants. The nanoemulsions are composed of oils, surfactants, or their mixture and aqueous phase. The oil phase is used as a solubilizer for hydrophobic drugs [57]. For the delivery of the hydrophobic drugs via NE, the drugs are incorporated into the oil phase to form nanodroplets dispersed into the continuous aqueous phase and provide an oil in water (O/W) NE system [58]. It was further observed that the rate of drug release from the NE primarily depends on the oil/water partition coefficient of the drug molecule, oil/lipidic content as well as the water content of the NE system [28]. In NE, 5–20 wt% of the oil or lipid is considered as the dispersed phase. Hence, screening of oil or lipids is proven to be an important aspect of nanoemulsion formation, as the API must get freely solubilized in the oils/lipids before their dispersion to the aqueous phase. The various oils/lipids used for the lipid phase are glycerides, medium-chain triglycerides, long-chain unsaturated fatty acids, vegetable oils, and polyalcohol esters of medium-chain fatty acids [59]. It was observed from various studies that the nanoemulsions developed using long-chain triglycerides (LCT) showed an average particle size of 120 nm, while those prepared using short-chain triglycerides (SCT) showed smaller particle sizes (40 nm), compared to the former ones. In addition, the lipidic core of the nanoemulsion exhibits an impact on drug loading, stability, and physicochemical attributes. The surfactant or the mixture of surfactants and co-surfactants is employed to reduce the interfacial tension between the lipid phase and aqueous phase for the development of a thermodynamically stable monophasic system. Moreover, it was found apart from reducing the surface tension, a suitable surfactant stabilizes the interfacial surface via electrostatic interactions. The various emulsifying agents used in nanoemulsion preparation include surfactants such as Tween 80, sodium dodecyl sulfate, phospholipids such as soy lecithin, zwitterionic proteins such as caseinate, polysaccharides such as modified starch, and polymers such as PEGs [60]. Various studies showed that the small size, large surface area, and tunable surface characteristics help the nanoemulsion in increasing their circulation half-life, and specific targetability towards cancer sites. As cancer cells are fenced by leaky vasculature, the nanoemulsions can easily bypass the physiological barriers and accumulate within the cancer cells [61]. Various anticancer drugs such as tamoxifen and dacarbazine have been administered in the NE system [28].

**Figure 4:**
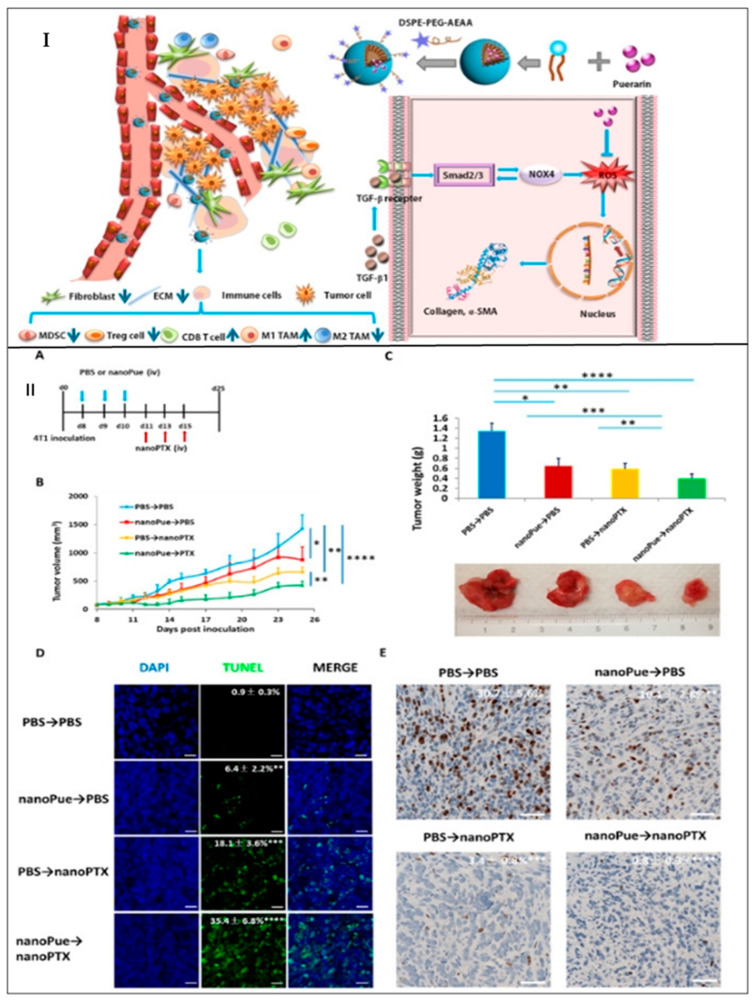
(**I**) Schematic diagram of TME remodulation by targeted delivery of puerarin-loaded nanoemulsion. (**II**) Combination treatment of nanoPue and nanoPTX on 4T1 tumor model: (**A**) nanoPue and nanoPTX combination treatment scheme. (**B**) Tumor growth curves of 4T1 tumors in different treatment groups. (**C**) The tumor weight and the representative tumor image at the end of the experiment in different treatment groups. (**D**) TUNEL staining of differently treated 4T1 tumor tissues. (**E**) Comparison of Ki67 expression of 4T1 tumors in different treatment groups. Scale bar represents 20 μm * *p* < 0.05, ** *p* < 0.01, *** *p* < 0.001 and **** *p* < 0.0001. Reprinted from Biomaterials, 235, Xu, et al., Nano-puerarin regulates tumor microenvironment and facilitates chemo- and immunotherapy in murine triple negative breast cancer model, 1-12, Copyright (2020), with permission from Elsevier [63].

Kim et al., 2019 developed decitabine (DAC)- and panobinostat (PAN)-loaded nanoemulsion which was further coated with lysophophatidylcholine and lysophophatidic acid for targeting LPC receptor and LPAR1 receptor that is overexpressed in TNBC cells. It was observed that the DAC-PAN-LNEs restored CDH1/E-cadherin and suppressed the expression of FOXM1 which eventually inhibited the growth of TNBC cells. Further, it was observed that DAC/PAN LNEs decreased the cell viability of MDA-MB-231 by 55% which indicated the increased therapeutic activity of NEs against TNBC. In addition, DAC/PAN-LNEs synergistically decreased the expression of FOXM1 mRNA and FOXM1 protein expressions by 80% [62]. Xu et al., 2020 developed puerarin nanoemulsion (NanoPue) using soya lecithin and Kolliphor^®^ HS15 for increased oral bioavailability and therapeutic efficacy against TNBC. It was observed that the NanoPue reduced the expression of tumor-associated fibroblast (TAFs) and enhanced the intra-tumoral infiltrations (ITLs) of cytotoxic T cells by 6-fold and 2-fold, respectively, as compared to control, and mediated chemotherapy effect of nano-paclitaxel in the desmoplastic triple-negative breast cancer (TNBC) model. Such an activated immune-microenvironment caused by NEs treatment facilitated a synergistic PD-L1 blockage approach for the treatment of TNBC (Figure 4) [63]. Han et al., 2021 fabricated elemene nanoemulsion (E-NE) for the treatment of TNBC as well as to inhibit their metastasis to the lung. The lipidic phase is comprised of soybean phospholipids and cholesterol. It was observed that E-NE reduced the stabilization of HIF-1α by effectively scavenging ROS. Additionally, the E-NE limited angiogenesis and NLRP3 inflammasomes and IL-1β [64]. Saraiva et al., 2021 developed edelfosine nanoemulsion (ET-NEs) containing lipids such as miglyol 812 and phosphatidylcholine. It was observed that the ET-NEs decreased tumor growth in vitro and in vivo. Furthermore, it was observed that the ET-NEs penetrated the physiological barriers of MDA-MB 231 xenografted zebrafish embryos, resulting in a significant reduction of cancer cell proliferation, which was further confirmed by confocal laser microscopy. Further, it was observed that the ET-NEs showed a dose-dependent IC_50_ which was found to be 6.9 μg/mL at 13.2 μM after 24 h of incubation, whereas the free ET showed a higher IC_50_ which is 13.9 μg/mL at 26.5 μM [65].

### 2.3. Solid Lipid Nanoparticles (SLNs)

Solid lipid nanoparticles (SLNs) are O/W type of colloidal nanoparticles consisting of an inner lipid-based phase and outer aqueous-based phase that are stabilized by surfactants or a mixture of surfactants [66,67]. It was observed from various studies that SLNs can either solubilize lipophilic chemotherapeutics homogeneously within the lipidic matrix or develop a drug-enriched shell surrounding the lipidic core [68,69]. Further, it was observed that based on the type of drug deposition pattern within the lipid matrices, i.e., either chemotherapeutics-enriched core or chemotherapeutics-enriched shell, the drug release profile can be regulated to our advantage for the fulfillment of desired release profile [70]. For example, SLNs with drug-enriched shells exhibit a biphasic drug-release profile through initial burst release from the outer shell followed by gradual release from the lipid core. However, in SLNs with drug-enriched core, a more prolonged sustained release profile was observed due to enhanced drug diffusional distance from the lipidic core [28]. The lipid-based phase used is the solid lipids including triglycerides, fatty acids, steroids, and waxes [71]. It was further reported that the lipids and surfactants used for the preparation of SLNs should fall under GRAS (Generally Recognized as Safe) regulations. The most widely used solid lipids are palmitic acid, stearic acid (SA), glyceryl monostearate (GMS), compritol 888 ATO, trimyristin, Capmul^®^MCM C10, soybean lecithin, etc. In terms of structure, SLNs show similarity with emulsion except for the fact that the SLNs replaced the oily core with a lipid-based core [8]. Moreover, the SLNs also show a certain amount of similarity as well as dissimilarity with the conventional liposomes. It was observed that like liposomes, SLNs are composed of lipids and unlike liposomes, the SLNs do not have a lipidic bilayer instead composed of a micelle-like structure. It was stated that lipids used in the SLNs should remain in solid form at room temperature and body temperature [3]. Such characteristics help in encapsulating lipophilic drugs in the melted lipid phase which further help in increasing the drug-loading capacity of SLNs and altering the physicochemical properties of drugs both in vitro and in vivo [72,73]. Such features also aid in reducing the degradation profile of the lipids making them suitable for the fabrication of the controlled release formulation. Rheologically, it was observed that the formation of SLNs depends on the interfacial tension (adhesive forces) between the two phases, where the addition of one or more surfactants the interfacial tension by reducing the surface energy and facilitates the formation of stable SLNs [72]. From various studies, it was observed that typically, SLNs are comprised of 0.1–30% solid lipid and 0.5–30% surfactant or surfactant blend [74]. SLNs exhibit increased loading capacity, entrapment efficiency, and less toxicity as compared to polymeric nanoparticles. Therapeutically, SLNs show enhanced targetability to cancer cells via passive targeting as well as active targeting with some external modifications. Additionally, SLNs could encapsulate various moieties such as drugs, proteins, nucleic acids, etc., and improve their pharmacokinetic attributes and physicochemical stability [71]. SLNs have been fabricated for the delivery of various lipophilic chemotherapeutics such as camptothecin, all-trans retinoic acid, etc. Despite the advantages provided by the SLNs, they do exhibit certain physical instability upon storage. It was observed that the solid lipids undergo crystallization during storage which limits the movement of active moieties within the lipidic core, mediating an expulsion of active moieties into the dispersion media and affecting the entrapment efficiency of the lipid-based nanosystem [66].

Eskiler et al., 2018 prepared BMN-673 loaded SLNs using GMS as solid lipids and Tween 80 as a surfactant to improve its therapeutic index and to overcome the BRCA1 mutated sensitive and resistant TNBC. It was observed that compared to native BMN 673, BMN 673-SLNs showed a significant decrease in HCC1937 and HCC1937-R cells with less damage to TNBC cells. In addition, BMN 673-SLNs induced significant toxicity in TNBC cells via breaking of double-stranded DNA, arresting of G2/M cell cycle, and cleaving of PARP moieties [75]. Siddhartha et al., 2018 developed di-allyl-disulfide (DADS)-loaded solid lipid nanoparticles using palmitic acid as solid lipid and pluronic F-68 and soy lecithin as surfactant mix, which was further conjugated with RAGE antibody to enhance the targetability and delivery of DADS to TNBC cells. It was observed that the DADS-RAGE-SLNs significantly increased the cytotoxicity and apoptosis (61.8%) as compared to DADS (15%). Additionally, DADS-RAGE-SLNs showed enhanced cellular internalization via receptor-mediated endocytosis as the SLNs bypassed P-gp efflux proteins as compared to DADS [76]. Kothari et al., 2019 fabricated docetaxel (DTX)–alpha-lipoic acid (ALA) co-loaded SLNs using GMS, SA, and Compritol ATO 888 as solid lipids and Tween 80 as a surfactant to treat TNBC. It was observed that the DTX-ALA-SLNs showed increased cytotoxicity to 4T1 cells as compared to DTX-SLNs, ALA-SLNs, and free drugs. Moreover, the DTX-ALA SLNs showed increased apoptosis of 32% as compared to free DTX which is only 11% [77]. Pindiprolu et al., 2019 prepared niclosamide-loaded SLNs (Niclo-SLNs) using stearyl amine as solid lipid and Tween 80, and pluronic F-68 as surfactant mix for the treatment of TNBC. It was observed that the Niclo-SLNs showed increased cytotoxicity and enhanced cellular internalization (77.06%) at the G0/G1 phase of the cell cycle as compared to free Niclo (69.50%). It was inferred that Niclo-SLNs showed increased cellular uptake due to their ability to bypass the efflux pump and increased absorption of drugs within the cancer cells [78]. In this context, Pindiprolu et al., 2020 fabricated phenylboronic acid-modified Niclo-SLNs (PBA-Niclo-SLNs) to enhance the targetability of Niclo to TNBC cells, thereby increasing its therapeutic efficacy toward TNBC cells. It was observed that PBA-Niclo-SLNs showed increased cytotoxicity (CTC50 7.311 ± 2.1 μM), inhibition of cell proliferation at G0/G1 cell cycle (74.01 ± 0.60%) and apoptosis (21.3 ± 1.0%) as compared to Niclo-SLNs (CTC50 18.49 ± 2.5 μM; 61.01 ± 1.10%; 12.3 ± 1.1%), and free Niclo (CTC50 31.17 ± 3.2 μM; 54.21 ± 0.90%; 10.8 ± 0.9%), respectively. Additionally, PBA-Niclo-SLNs significantly inhibited STAT3, TNBC stem cell populations (CD44+/CD24−), and EMT (epithelial–mesenchymal transition) markers along with increased tumor-site accumulation with significant tumor regression and enhanced survivability of TNBC-bearing mice [79]. 

### 2.4. Nanostructured Lipid Carriers (NLCs)

Nanostructured lipid carriers (NLCs) are considered second-generation lipid-based nanoparticles. It is composed of a mixture of solid lipid and liquid lipid which is further stabilized in the aqueous phase via one or more surfactants [80]. The incorporation of liquid lipid and solid lipid within the lipid matrix forms a massive crystal imperfection or amorphous structure that facilitates enhanced loading of drugs into the lipid matrix with less pronounced drug expulsion [8,74]. It was further observed that the NLCs obtained should remain solid at a temperature higher than 40 °C. Generally, NLCs encapsulate approximately 5% of drug *w/v* where approximately 3 to 4% drug loading is obtained (entrapment efficiency of ≈70%) [74]. The various solid lipids employed in NLCs preparation are glyceryl tripalmitate, softisan 154, glyceryl monostearate, compritol ATO 888, stearic acid, precirol, PEG-DSPE, soybean phosphatidylcholine, etc., and the liquid lipids used for the preparation of NLCs include glyceryl tridecanoate, olive oil, labrafil WL 2609 BS, oleic acid, labrafil M2125 Cs, labrafac PG, polyoxyl castor oil, etc. From various studies, it was observed that the NLCs show high tolerability due to the existence of lipids that are bio-compatible and tunable. Such characteristics enable NLCs to show increased drug loading capacity, decreased risk of gelation, and restricted leakage of the drug upon storage. In addition to these, NLCs also extend their exposure period over tumor cells via the EPR effect, thereby increasing the therapeutic efficacy of the antitumor drug on the tumor site [8]. The NLCs are further multi-functionalized to increase the drug payload, increase targetability to the cancer site, and release the drug in a more controlled way [74]. 

**Figure 5:**
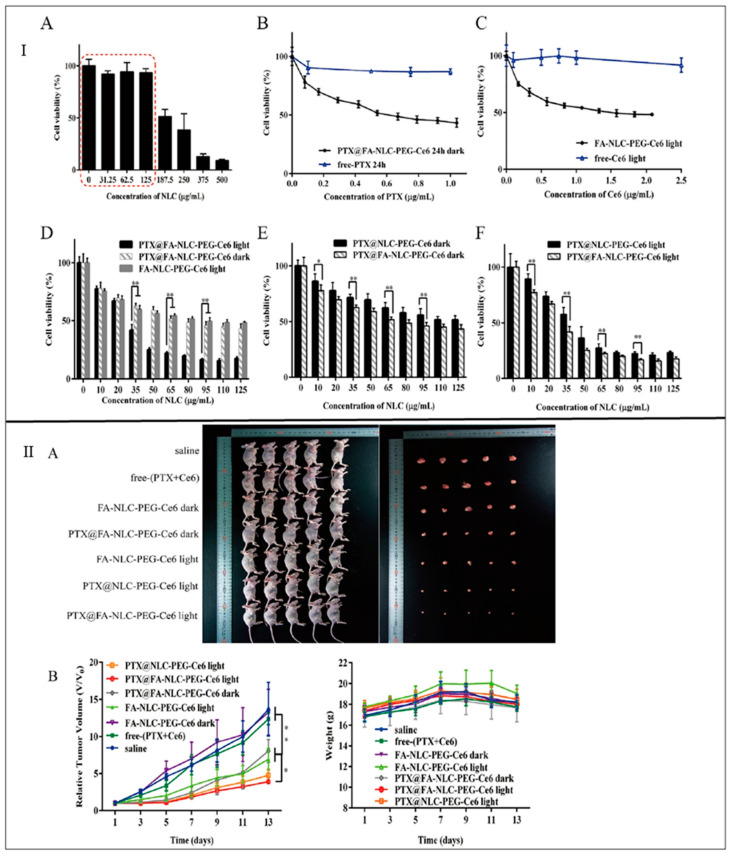
(**I**) In vitro cytotoxicity assays: (**A**) Cell viability of MDA-MB-231 cells after incubation with FA-NLC-PEG-Ce6 (dark) NPs of different concentrations. (**B**) Cytotoxicity evaluation of free-PTX and PTX@FA-NLC-PEG-Ce6 in dark in MDA-MB-231 cells by MTT. (**C**) Cytotoxicity evaluation of free-PTX and PTX@FA-NLC-PEG-Ce6 in light in MDA-MB-231 cells by MTT. (**D**) Cytotoxicity comprehensive evaluation of single drug NLCs and combination drug NLCs in MDA-MB-231 cells by MTT. (**E**) Cytotoxicity evaluation of PTX@NLC-PEG-Ce6 and PTX@FA-NLC-PEG-Ce6 in MDA-MB-231 cells with red laser by MTT. (**F**) Cytotoxicity evaluation of PTX@NLC-PEG-Ce6 and PTX@FA-NLC-PEG-Ce6 in MDA-MB-231 cells without red laser by MTT. * *p* < 0.05, ** *p* < 0.01. (**II**). In vivo anti-cancer activity of NLCs in tumor-bearing nude mice after intravenous administration of saline, free (PTX + Ce6) and different kinds of NLCs, with red laser after 24 h of injection (each mouse for 30 min): (**A**) Photographs of sacrificed nude mice and the tumor tissues collected from them. (**B**) Changes of relative tumor volumes in MDA-MB-231 tumor-bearing nude mice of each group. Note: * *p* < 0.05, ** *p* < 0.01 (*n* = 5). Reprinted from International Journal of Pharmaceutics, 569, Zhang, et al., Construction and in vitro and in vivo evaluation of folic acid-modified nanostructured lipid carriers loaded with paclitaxel and chlorin e6, 1-12, Copyright (2019), with permission from Elsevier [82].

Pedro et al., 2019 prepared paclitaxel-loaded NLCs (PTX-NLCs) using compritol ATO 888 as solid lipid and MCT as liquid lipid to increase its therapeutic efficacy against TNBC. The NLCs were further stabilized by using Tween 80 and soya lecithin. It was observed that the PTX-NLCs showed increased in-vitro cell cytotoxicity and anti-clonogenic activity against MDA-MB-231 cells as compared to free PTX. Further, from the cell viability assay, it was observed that the free PTX showed more cell viability which is 56.0 ± 3.2% as compared to PTX-NLCs (38.0 ± 5.0%). Additionally, PTX-NLCs exhibited 1.5- and 1.7-fold increased tumor site accumulation after 30 and 120 min, respectively, in tumor-bearing mice, as compared to free PTX [81]. Zhang et al., 2019 fabricated folic acid (FA)-functionalized paclitaxel (PTX) and chlorin e6 (Ce6)-loaded NLC (PTX-Ce6-NLC) to increase their targetability and therapeutic efficacy against TNBC. The NLCs were prepared using Precirol ATO 5 as solid lipid and Maisine 35-1 as liquid lipid, stabilized by Cremophor RH40. It was observed that FA-PTX-Ce6-NLC showed enhanced MDA-MB-231 cellular uptake via FR-mediated endocytosis as compared to free PTX. Moreover, it was observed that Ce6 dissociated and evenly distributed in tumor cells. Additionally, from the pharmacodynamic study, it was observed that the NLCs showed enhanced drug-loading without side effects as compared to free PTX (Figure 5) [82].

Lages et al., 2020 developed doxorubicin- and α-tocopherol succinate-loaded NLCs using compritol 888 ATO as solid lipid and docosahexaenoic acid (DHA) as liquid lipid to increase their anti-cancer activity against TNBC. Tween 80 was employed as a surfactant to further stabilize the lipid phased in aqueous media. It was observed that the NLCs showed a controlled release profile with an increased release in acidic media. Further, the NLCs showed decreased mortality in mice, reduced metastasis to lungs, and prevented drug-induced toxicity to vital organs (heart and liver) as observed from biochemical and histological assays. In addition, the NLCs showed a higher tumor inhibition ratio (76.6%) as compared to free doxorubicin (64.6%) [83]. Gadag et al., 2021 prepared resveratrol-loaded NLCs (RVT-NLC) using GMS as solid lipid and caproyl 90 as liquid lipid for increasing the therapeutic efficacy of resveratrol against TNBC. The NLCs were stabilized using labrasol as a surfactant. From the cell viability study, it was observed that the RVT-NLCs showed decreased MDA-MB-231 cell-viability (IC_50_ = 27.50 ± 3.43 μg/mL), as compared to free RVT (IC_50_ = 33.93 ± 7.34 μg/mL), which indicated that the RVT-NLCs were found to be more potent as compared to free RVT. Further, to increase the therapeutic efficacy via the dermal route, the RVT-NLCs were loaded within microneedle. It was observed that the RVT-NLCs loaded microneedle showed increased skin permeation, improved cellular internalization, and prevented metastasis as compared to free RVT. In addition, the RVT-NLCs increased pharmacokinetic attributes (C_max_ = 343.75 ± 31.89 ng/mL; AUC_0-t_ = 4529.2 ± 299.67 h∗ng/mL), as compared to free RVT (C_max_ = 269.30 ± 30.26 ng/mL; AUC_0-t_ = 458.3 ± 21.21 h∗ng/mL) [84]. Gilani et al., 2021 prepared luteolin-loaded NLCs (LTN-NLC) using GMS as solid lipid and caproyl 90 as liquid lipid to treat TNBC, stabilized by poloxamer 188. Further, to obtain a sustained release profile, the NLCs were surface functionalized by chitosan (LTN-CS-NLCs). It was observed that LTN-CS-NLCs exhibited a slow-release profile of LTN during a 24 h study. Moreover, LTN-CS-NLCs showed increased mucoadhesion, improved gastrointestinal stability, and intestinal permeation as compared to free LTN. In addition, from the MTT assay, it was observed that LTN-CS-NLCs showed decreased MDA-MB-231 cell viability (IC_50_ = 11.48 ± 2.38 μM), as compared to free LTN (IC_50_ = 29.64 ± 3.84 μM) after 48 h treatment. Additionally, LTN-CS-NLCs exhibited 4.3-fold increased intestinal permeation as compared to LTN suspension, indicating the superiority of NLCs in overcoming P-gp efflux pump-mediating elimination, in comparison to suspension [85].

### 2.5. Lipid Polymer Hybrid Nanoparticles (LPH-NPs)

Lipid–polymer hybrid nanoparticles (LPH-NPs) are considered new-generation nanoparticles exploiting the advantages of both polymeric nanoparticles and lipid-based nanoparticles in a single nanosystem [86]. Such a system is comprised of a polymeric core surrounded by a lipidic monolayer, which could be further surface-functionalized via PEGylation to prolong its circulation or via ligand to enhance its targetability. LPH-NPs impart certain characteristics of lipid-based nanoparticles which include enhanced drug-loading capacity, biodegradable and biomimetic nature, and certain traits of polymeric nanoparticles such as controlled/sustained drug release profile and a variety of surface functionalization or modification. The various polymers used in the preparation of LPH-NPs are approved by the Food and Drug Administration (FDA) which include polycaprolactone (PCL), poly (lactic-co-glycolic acid) (PLGA), polylactic acid (PLA), poly β-amino ester (PbAE), etc. In terms of lipidic components, charged or zwitterionic lipids are selected as they could be exploited to mediate covalent or non-covalent bonds with the desired ligands, antibodies, and nucleic acids (DNA, RNA), proteins, or peptides. Moreover, the presence of charged moieties helps to facilitate electrostatic interaction between lipids and oppositely charged polymers (core), which would result in the development of self-assembling nanostructures. The various lipids used are DOTAP/DOPE, stearic acid, cholesterol, lecithin, lipoid GmbH, DSPE-PEG, and PEG2000-Mal [44]. It was further observed that the lipidic monolayer acts as a molecular barricade that alleviates the loss of loaded drugs throughout the LPH-NP preparation and safeguards the polymeric core from deterioration by inhibiting the diffusion of water into the polymeric core. Due to the hybrid nanostructure, LPH-NPs can incorporate anticancer drugs of different physicochemical profiles. They are also able to conjugate ligands that are overexpressed on cancer cells, resulting in enhanced targetability and therapeutic efficacy with restricted off-site toxicities. In addition, LPH-NPs exhibit improved mechanical stability upon storage. Further, it was observed that the LPH-NPs of dimensions ≤ 100 nm display increased intra-cellular release of drug(s), resulting in decreased cytotoxicity.

**Figure 6:**
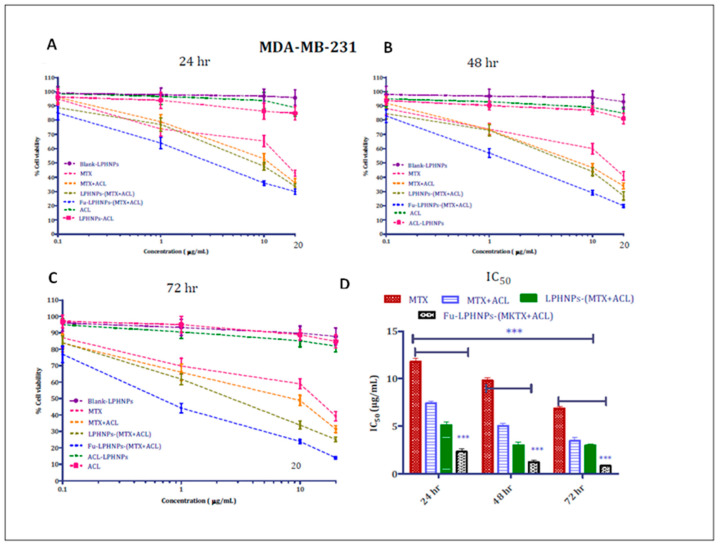
The in vitro anti-cancer activity of MTX- and ACL-based free and co-encapsulated LPHNPs after 24 (**A**), 48 (**B**), and 72 h (**C**) in MDA-MB-231 cell lines. The image (**D**) depicts the comparison of IC_50_ of different samples obtained after 24, 48, and 72 h (*** *p* < 0.05). The statistics were run to determine significance in IC_50_ by 2-way ANOVA, and data are presented as the mean of three independent experiments (SD, *n* = 6). Reprinted (adapted) with permission from [89]. Copyright (2017) American Chemical Society.

Zhang et al., 2017 prepared RGD-conjugated doxorubicin (DOX) and mitomycin C (MMC) co-loaded lipid–polymer hybrid nanoparticles (DMPLN) to treat TNBC. In the preparation of DMPLN, HPESO (hydrolyzed polymer of epoxidized soyabean oil) was used as a polymeric core and myristic acid was used as a lipid layer. It was observed that the RGD-DMPLN facilitated certain morphological changes and induced cytotoxicity. Moreover, compared to free drugs, RGD-DMPLN showed increased cellular accumulation, restricted lung metastasis (31-fold), remarkably decreased toxicity to the liver and heart, and improved median survival time (57%) [87]. Zhou et al., 2017 formulated calcium-phosphate-based lipid–polymer hybrid nanoparticles (LPH-NPs) co-loaded with paclitaxel and inhibitors of microRNA-221/222 for the treatment of TNBC. The LPH-NPs were prepared by using PLGA and PEG as polymers and dioleoylphosphatidic acid (DOPA) as anionic lipid. It was observed that the cell viability got decreased by approximately 80% in the case of co-loaded LPH-NPs as compared to free paclitaxel at the same dose of 0.67 μg/mL. Additionally, the co-loaded LPH-NPs showed enhanced intracellular activity as compared to free paclitaxel [88]. Garg et al., 2017 prepared fucose-anchored lipid–polymer hybrid nanoparticles (LPH-NPs) co-loaded with methotrexate (MTX) and aceclofenac (ACL) for the treatment of TNBC. LPH-NPs were prepared using gelucire 48/16 (lipid layer), phospholipid 90NG, and phospholipid S100 (polymer layer). Further, the LPH-NPs were conjugated with DSPE-PEG (2000)-NH-gructose. It was observed that LPH-NPs exhibited rapid MDA-MB-231 cellular internalization within 2 h and showed 10-fold increased bioavailability as compared to free drugs. Additionally, LPH-NPs showed ~21–25% less MDA-MB-231 cell growth, and 5–6 times increased mean residence time (MRT) as compared to free drugs (Figure 6) [89]. Bakar-Ates et al., 2020 developed cucurbitacin B-loaded lipid polymer hybrid nanoparticles (CuB-NPs) by using polymers such as PLGA, DSPE-PEG, and lipids such as lecithin for the treatment of TNBC by inducing apoptosis in MDA-MB-231 cells. It was observed that the CuB-NPs showed decreased cell viability at 0.1 and 5 μM concentrations as compared to the control. Additionally, the treatment with CuB-NPs showed increased cell population at G0/G1 phase (56.50 ± 1.23%), and apoptosis (20.66 ± 1.99%) as compared to free CuB (47.20 ± 1.02% and; 3.69 ± 0.57%, respectively) [90].

### 2.6. Exosomes

Exosomes are nanosized extracellular vesicles that are enclosed by lipidic bilayers with diameter ranging from 30–150 nm, and are released by almost all kinds of cells [39]. In this context, our lab has isolated exosomes from bovine milk with particle size of 75 ± 0.6 nm [91,92], and colostrum with particle size 59 ± 1.1 nm [93]. The isolated exosomes further exhibited an increased therapeutic efficiency of anticancer agents to the targeted site [94,95]. They are basically generated by two invaginations of the plasma membrane. Exosomes are comprised of various surface proteins that are specific to the endosomal pathway, and can enclose nucleic acid, receptors, cytosolic proteins, and drugs [96,97]. The lipidic layer of exosomes varies from other types of extracellular vesicles such as apoptotic bodies and microvesicles as they are enriched in cholesterol and diacylglycerol [45,98]. Exosomes are considered one of the encouraging natural carriers of antineoplastics or biomolecules as they bypass their elimination through circulation and enhance cell-specificity towards cancer cells after modification via surface proteins [99]. It was observed that exosomes are biodistributed via body fluids to transport drugs or biomolecules to the cancer cells within the vicinity or dwelling remotely, which offers an advantage in recognizing potential pathological situations. The exosomes follow various uptake mechanisms namely direct membrane fusion, or endocytosis [100].

**Figure 7:**
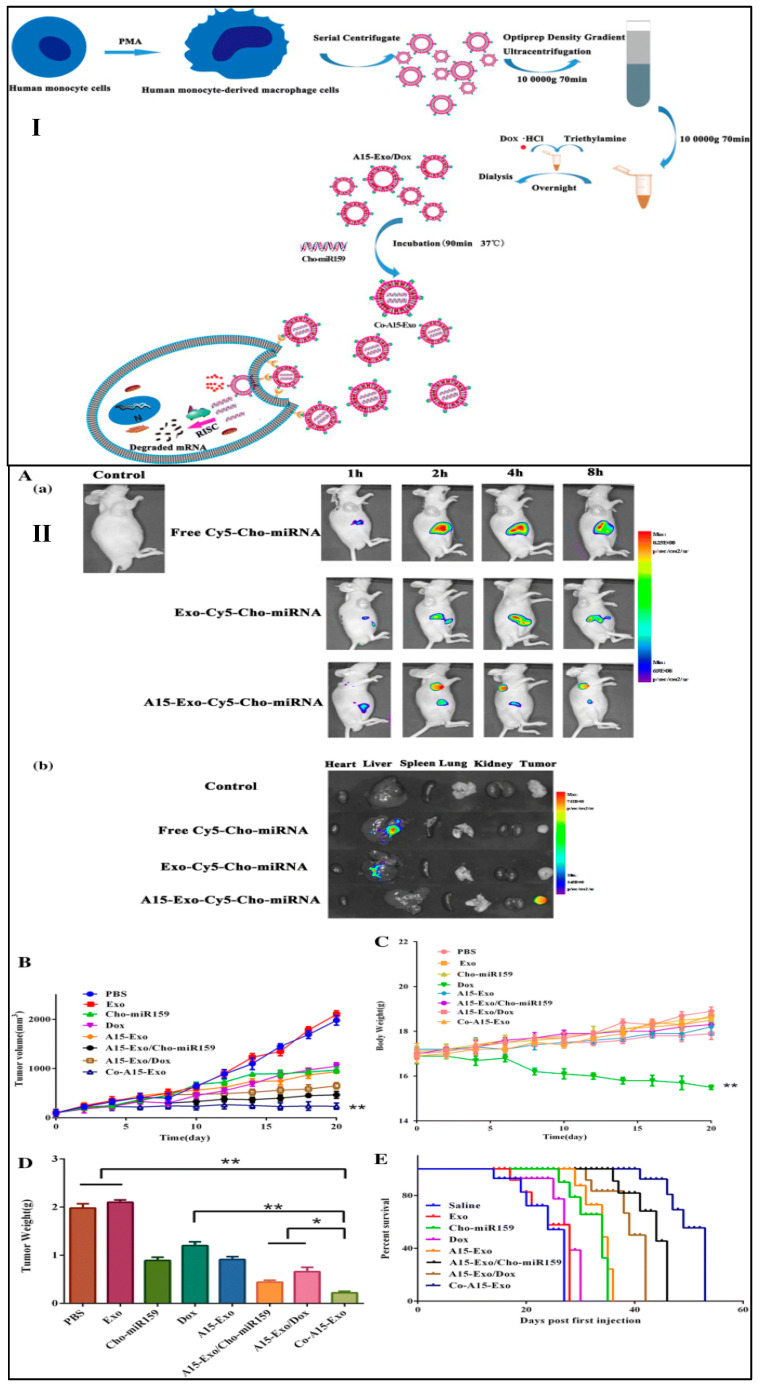
(**I**) Schematic diagram of isolation of exosomes, loading of Dox.Hcl and Cho-miR159 within the exosomes and release of Dox and Cho-miR159-loaded A15-Exo (Co-A15-Exo). (**II**) Biodistribution and antitumor efficacy of Co-A15-Exo in vivo: (**A**(**a**)). Images were taken 1 h, 2 h, 4 h, or 8 h after the administration of free Cy5-Cho-miRNA, Exo-Cy5-Cho-miRNA, or A15-Exo-Cy5-Cho-miRNA. (**A**(**b**)) Ex vivo imaging of tumor and organs collected at the end of the experiment (8 h post-injection). (**B**) Tumor growth curves of mice receiving different therapeutic regimens (*n* = 5, mean ± SD). (**C**) Body weight changes during treatment. Data are expressed as the mean ± SD (*n* = 5). ** *p* < 0.01, vs. PBS. (**D**). The weights of the excised tumor tissues from all groups. Data are expressed as the mean ± SD (*n* = 5). * *p* < 0.05 and ** *p* < 0.01 when compared with the indicated groups. (**E**) Survival rate of MDA-MB-231 tumor-bearing BALB/c nude mice [102].

Naseri, et al., 2018 isolated exosomes from bone marrow-derived mesenchymal stem cells, loaded with locked nucleic acid (LNA)-modified anti-miR-142-3p oligonucleotides (MSCs-Exo) to diminish the expression of miR-142-3p in 4T1 breast cancer cell lines. It was observed from the in vitro and in vivo results that the MSCs-Exo showed efficient delivery and enhanced penetration of anti-miR-142-3p in breast cancer cells, respectively, along with increased transcription of regulatory target genes [101]. Gong et al., 2019 isolated exosomes from human leukemia monocytic cell line (THP-1), co-loaded with doxorubicin hydrochloride (Dox), and cholesterol-modified miRNA (Cho-miR159) to treat TNBC. Further to increase the targetability of co-loaded exosomes, the system was further conjugated with modified version of a disintegrin and metalloproteinase 15 (Co-A15-Exo) (Figure 7I). It was observed that flow cytometry data that the A15-Exo exhibited increased cellular uptake (78.60%), as compared to Exo (15.23%). Moreover, Co-A15-Exo showed enhanced apoptosis in MDA-MB-231 cells as compared to Dox-treated group. Further, Co-A15-Exo showed increased inhibitory rates of tumor volume (92.8%), as compared to Dox (49.5%), and Cho-miR159 (53.7%), revealing a potent synergism among Dox and Cho-miR159 (Figure 7II) [102]. Yu et al., 2019 fabricated erastin-loaded HFL-1 (human fetal lung fibroblast) derived exosomes conjugated with folic acid (FA) to target TNBC cells with overexpressed FA receptors (Erastin@FA-exo). It was observed that Erastin@FA-exo increased the cellular uptake of erastin into MDA-MB-231 cells, compared to free erastin. In addition, Erastin@FA-exo exhibited significant inhibition of TNBC cells proliferation and migration and promoted ferroptosis along with depletion of intracellular glutathione and ROS production [103]. Li et al., 2020 developed c-Met binding protein conjugated engineered exosomes for the treatment of TNBC. The author developed doxorubicin (Dox)-loaded polymeric nanoparticles and incorporated them into the macrophages-derived exosomes. It was observed that the engineered exosomes exhibited increased cellular uptake of Dox (2.28 times and 3.31 times), as compared to free-Dox and Dox-loaded polymeric nanoparticles, respectively. Further, the engineered exosomes showed increased apoptosis rate (39.73%), as compared to free Dox (10.58%) and Dox-loaded polymeric nanoparticles (11.33%) [104]. In this context, it is a noteworthy development that our lab also developed paclitaxel- and 5-fluorouracil-loaded exosomes, isolated from bovine milk and surface conjugated with folic acid for offering an effective treatment regimen against breast cancer. It was observed that developed exosomes showed an average particle size of 80–100 nm, with 82% entrapment efficiency. Moreover, the surface functionalized loaded exosomes showed increased cellular uptake and higher apoptotic index, compared to free drugs [97]. 

The various LNPs fabricated in the last decade to enhance therapeutic efficacy against TNBC have been summarized in Table 3.

## 3. Clinical Status

Liposomes have been employed in clinical applications for many years. From the survey, it was observed that there are approximately 21 approved liposomal formulations, out of which Doxil first reached clinics and opened the door for other nanoformulations by FDA viz. Myocet^®^, Lipodox^®^, and liposomal doxorubicin for the treatment of breast neoplasms, where Myocet^®^ is the conventional liposomal formulation, whereas Doxil^®^, Lipodox^®^, and Doxorubicin are the PEG-conjugated liposomes, otherwise called stealth liposomes. For the treatment of solid tumors and acute lymphoblastic leukemia, the FDA has approved another liposomal formulation of vincristine named Marqibo^®^. As cancer research progresses, the concept of combination therapy has evolved and is subject to much attention, as compared to monotherapy. Taking this into account, Vyxeos^®^ was formulated and approved in 2017 for the effective treatment of acute myeloid leukemia, as it provided the synergistic anticancer activity of daunorubicin and cytarabine [105]. The various LNPs approved by FDA are shown in Table 4.

Various other liposomes are under clinical trials, namely ThermoDox^®^, a thermosensitive liposome encapsulating doxorubicin. As it is well known that the tumor microenvironment experiences increased temperature than the usual body temperature, ThermoDox^®^ utilizes this concept and releases the drug at the tumor site that experiences a higher temperature (>40 °C), hence increasing the targetability of the anticancer drug. Such formulation was fabricated for the treatment of liver cancer. ThermoDox^®^ procured US FDA Fast Track Designation and was granted orphan drug designation in both the US and Europe for the treatment of primary liver cancer [106]. Recently, ThermoDox^®^ completed a phase III trial (NCT02112656), where ThermoDox^®^ is combined with standard radiofrequency ablation [105]. While the liposomes are occupied in clinical studies, the other LNPs are still under pre-clinical trials (in-vitro and in-vivo), as presented in Table 5.

**Table 4:** FDA-approved LNPs for various diseases including cancer.

S.No.	Brand Name	Formulation	Company of Manufacture	Use	Approval Year	Ref.
1	Doxil	Liposomal doxorubicin HCl (PEGylated)	Janssen	Kaposi’s sarcoma, ovarian cancer, multiple myeloma	1995	[107]
2	DaunoXome	Liposomal daunorubicin	Galen	Kaposi’s sarcoma	1996	
3	DepoCyt©	Liposomal cytarabine	Pacira Pharms Inc.	Lymphoma	1996	
4	Myocet	Liposomal doxorubicin (non-PEGylated)	Teva UK	Metastatic breast cancer	2000	[108]
5	MEPACT	Liposomal Mifamurtide	Takeda	Osteo-sarcoma	2009	[109]
6	Marqibo	Liposomal vincristine	Acrotech Biopharma	Acute lympho-blastic leukaemia	2012	[110]
7	Onivyde	Liposomal irinotecan	Ipsen	Metastatic pancreatic cancer	2015	[111]
8	Vyxeos	Liposome encapsulating Cytarabine: daunorubicin in fixed-dose	Jazz Pharmaceuticals	Acute myeloid leukemia	2017	[112]

**Table 5:** List of LNPs subjected to pre-clinical trials and clinical trials.

S.No.	Cancer Type	LNPs	Route; Size	Status	Ref.
1.	Glioblastoma	Curcumin-loaded NE	Oral; 67 ± 6 nm	In vitro and In vivo	[113]
		Transferrin conjugated liposome encapsulating doxorubicin and erlotinib	158.7–165.05 nm	In vitro	[114]
		naI- IRI loaded liposome targeting topoisomerase I	Intravenous; 88–95 nm	Phase I clinical trial	[115]
		Docetaxel-loaded SLN targeting LRP1	Intravenous; 79–111.4 nm	In vitro and In vivo	[116]
		Ferulic acid-loaded NLCs	<50 nm	In vitro	[117]
		Lactoferrin and RGD peptide conjugated NLCs encapsulating temozolomide and vincristine	Intravenous; 96 nm	In vitro and In vivo	[118]
2.	Esophageal	Rhenium loaded liposomes	Intravenous; <100 nm	In vitro and In vivo	[119]
		LY294002 and 5-FU co-loaded Liposome (PEGylated) targeting thymidylate synthase	Intravenous; 110 nm	In vitro and In vivo	[120]
3.	Lung	9-bromo-noscapine-loaded NE	Inhalation; 13.4 ± 3.2 nm	In vitro and In vivo	[121]
		Diferuloylmethane-loaded NE	Oral; ∼232.7 nm	In vitro and In vivo	[122]
		PEG-lecithin and nRGD peptide conjugated NE-loaded lycobetaine and oleic acid	Intravenous; 158.42 ± 2.87 nm	In vitro and In vivo	[123]
		Paclitaxel–Carboplatin–Gemcitabine-loaded liposome targeting tubulin	Percutaneous; 130 nm	Phase III clinical trial	[124]
		miR-34a conjugated Paclitaxel-loaded SLNs	Intravenous; 218.2 nm	In vitro and In vivo	[125]
		Transferrin-conjugated SLNs encapsulating Docetaxel and Baicalin	Intravenous; 135.5 nm	In vitro and In vivo	[126]
		Gemcitabine and Paclitaxel co-loaded NLC with surface functionalized via glucose receptor-targeting ligand	120.3 ± 1.3 nm	In vitro	[127]
4.	Breast	Doxorubicin and bromotetrandrine (W198)-loaded NE	Intravenous; 99.5–152.6 nm	In vitro and In vivo	[128]
		Doxorubicin and lapatinib-loaded liposome (PEGylated)	Intravenous; 100 nm	Phase Ib clinical trial	[129]
		Hyaluronic acid-coated Paclitaxel-pDNA-loaded SLNs	Intravenous; 156.3 ± 5.5 nm	In vitro and In vivo	[130]
		Fucose-conjugated Methotrexate-loaded SLNs	Intravenous; 174.51 ± 5.1 nm	In vitro and In vivo	[131]
		Lapachone and Doxorubicin loaded NLCs	Intravenous; 100.2 ± 6.8 nm	In vitro and In vivo	[132]
5.	Liver	Cantharidin-loaded liposomes (PEGylated)	Intravenous; 129.9 ± 2.5 nm	In vitro and In vivo	[133]
		Glycyrrhetinic acid-functionalized curcumin-loaded liposomes	Intravenous; 194 ± 0.25 nm	In vitro and In vivo	[134]
		miR-34a surface-functionalized liposomes	Intravenous; 120.21 ± 5 nm	Phase I clinical trial	[135]
		Sorafenib-loaded SLNs	248 ± 113 nm	In vitro	[136]
		Paclitaxel-loaded NLCs	Oral; 153.8 ± 5.58 nm	In vitro and In vivo	[137]
6.	Gastric	Indocyanine green-loaded liposome (PEGylated)	Intravenous; ~106 nm	In vitro and In vivo	[138]
		CD44 antibody-conjugated SATB1 siRNA-loaded liposome	159.3 nm	In vitro	[139]
		Etoposide-loaded SLNs	30–50 nm	In vitro	[140]
		Sorafenib and miR-542-3p-loaded SLNs (PEGylated)	Intravenous; ~156 nm	In vitro and In vivo	[141]
		Etoposide and curcumin co-loaded NLCs	Intravenous; 114 nm	In vitro and In vivo	[142]
7.	Pancreatic	Gemcitabine-loaded NE	~150 nm,	In vitro	[143]
		Gemcitabine and synthetic curcumin (EF24) combined loaded liposomes (PEGylated)	Intravenous; < 150 nm	In vitro and In vivo	[144]
		HSA-conjugated liposomes encapsulating Paclitaxel and Ellagic acid	Intravenous; 176.2 nm	In vitro and In vivo	[145]
		naI-IRI, 5-FU, and Leucovorin co-loaded liposomes	Intravenous; <200 nm	Phase III clinical trial	[146]
8.	Colorectal	Folic acid-conjugated 5-FU-loaded liposome	Intraperitoneal; 114 ± 4.58 nm	In vitro and In vivo	[147]
		Omega 3-fatty acid (DHA) and resveratrol-loaded SLNs	100 ± 1.8 nm	In vitro	[148]
		Folic acid and dextran-conjugated SLNs encapsulating Doxorubicin	Oral; 99–144 nm	In vitro and In vivo	[149]
		Hyaluronic acid-conjugated Irinotecan-loaded NLCs	386 ± 2.2 nm	In vitro	[150]
9.	Prostrate	Omega 3-fatty acid-conjugated Taxoid prodrug-loaded NE	Intravenous; 228 ± 7 nm	In vitro and In vivo	[151]
		Catechin extract-loaded NE	11.45 nm	In vitro	[152]
		Oleuropein-loaded liposome (PEGylated)	Intravenous; 184.2 ± 9.16 nm	In vitro and In vivo	[153]
		LRP1-targeted docetaxel-loaded liposome (PEGylated)	Intravenous; 163.2 ± 1.83 nm	In vitro and In vivo	[154]

From Table 5, it was observed that most of the LNPs employed in pre-clinical trials for the treatment of different types of cancers were mostly administered via intravenous route followed by oral and inhalation. Thus, if we just analyze the impact of LNPs over route of administration and targeting tumor site, based on the pre-clinical and clinical trials, we can conclusively state that for targeting lung cancer, pulmonary/inhalational route of administered was considered because the alveolar region of the lungs has larger surface area (~100 m^2^), extensive vasculature, thin alveolar epithelium (0.1–0.2 μm), and fewer drug-metabolizing enzymes, which allows enhanced absorption and bioavailability of nanosized LNPs loaded with chemotherapeutics. Moreover, the mucus membrane present within the alveolar region is composed of phospholipids (lipids) that are the major components of LNPs; as a result, LNPs are considered more biocompatible than other types of nanoparticles [155]. Similarly, brain targeting possesses a challenge for the delivery of hydrophilic chemotherapeutics, as they were unable to bypass blood–brain-barrier (BBB), hence LNPs were prepared to deliver hydrophilic drugs into the brain. It was observed that LNPs increased the lipophilicity of the drug which facilitates their transportation to brain by crossing the BBB. It was further observed that liposomes can cross BBB via receptor-mediated endocytosis (RMT), which facilitates enhanced accumulation of chemotherapeutics within tumor site. As a result, the off-target side effects were reduced. It was also observed from various studies that for the treatment of brain cancer, LNPs were administered by oral, intravenous as well as intranasal delivery [156]. The cellular uptake of LNPs by the oral route was already mentioned in Section 2. Briefly, on oral and intravenous administration, the LNPs enters the lymphatic and systemic circulation respectively, after which it targets the brain cancer cells via active (ligand-mediated cellular internalization) or passive (general cellular uptake via EPR effect) targeting [157]. Similar route and the associated approaches were employed for targeting breast, pancreas, and prostate cancer cells [24,158]. During intranasal administration for brain targeting, the LNPs binds with the mucus layer, which were then taken up by the neurons and translocated in the nerve axons to enter into brain cells, where the LNPs get degraded by the enzymes and drugs get released [156]. LNPs also ensure distinct drug delivery to the lesion site of the colon and rectum for the treatment of colorectal cancer. Most of the LNPs employed for the treatment of colorectal cancer are via oral route. It was observed that the LNPs are absorbed from the intestinal lumen into the circulation of colorectal region through endocytosis or via carrier-mediated transport [159]. Similarly, for treating hepatocellular carcinoma (liver cancer), LNPs are presently under pre-clinical and clinical trials. Like other cancer types, most of the chemotherapeutics are administered orally or intravenously for the treatment of hepatocellular carcinoma. It was observed that after administration of drug loaded LNPs, they non-specifically bind with the serum proteins leading to aggregation and opsonization, also causing a chance to get blocked within sinusoidal fenestrations. Therefore, to overcome the drawbacks, the LNPs were shielded with PEG, which provide shielding from plasma protein recognition as well as minimizing their sizes to <100 nm in order to cross the sinusoidal fenestrations. Moreover, liver targeting can be achieved by active or passive targeting [160]. 

The clinical achievement of LNPs with chemotherapeutics and nucleic acids revealed the potential of LNPs in the treatment of different types of cancer, However, the number of fruitful products reaching the market does not accurately represent the number of LNPs employed in pre-clinical trials, which indicated that the LNPs still suffers certain challenges while translating from animals to humans. Currently, various strategies have been developed to overcome such challenges. To further improve the stability and protect the drug from leaking, the lipidic structures have been modified that effectively form complex with the encapsulated chemotherapeutic via ionic attraction. Further stability of LNPs in systemic circulation was accomplished by PEGylating the LNPs, which safeguard them by reducing their recognition via RES. However, such approach leads to the production of anti-PEG antibodies which reduces the therapeutic efficacy of the LNPs. This incidence results in finding an alternative for PEGylation upon repeated administration. In addition to establish safety and therapeutic efficacy during prolonged circulation, the LNPs must also exhibit enhanced targetability and cellular internalization at the site of action [105]. To accomplish such objectives, the LNPs are fabricated with selective ligands which enables release of drug on targeted site when triggered by aberrations in pH, temperature, oxidation, or reduction within the tumor microenvironment [105,161].

## 4. Toxicity of Lipid-Based Nanoparticles (LNPs)

Despite the potential therapeutical efficacy, the LNPs do give a certain level of toxicity which includes cytotoxicity, and genotoxicity. It was observed from animal models that the presence of lipid-based nanoparticles activates the complement cascade leading to acute hypersensitivity reactions and anaphylaxis in almost 45% of patients. Further studies revealed that the toxicity of most of the FDA-approved nanoformulations is associated with their surface charge [162]. In this context, it was observed that cationic LNPs are therapeutically useless, as they activate immunological responses and inflammatory reactions. From previous studies, it was observed that LNPs are associated with 48–53% cytotoxicity in cell culture and animal models. Thus, to overcome such toxicities, the LNPs are encouraged to be fabricated in a solvent-free process employing only GRAS (Generally Recognized as Safe) approved ingredients. Lipid micelles, nanoemulsions, SLNs, and NLCs are considered well-tolerated LNPs as compared to liposomes. Orlando et al., 2013 experimented with SLNs and it was observed that the highest concentration of SLNs (1500 μg/mL) showed the highest concentration of NO in the macrophages, while in another study, it was observed that SLNs showed 10-fold and 100-fold less cytotoxicity as compared to poly-lactic acid nanoparticles (PLA NPs) and butyl cyanoacrylate nanoparticles (BC-NPs), respectively. Such findings revealed that the toxicity of LNPs is associated with the type and concentration of lipid used as a matrix. Herein, triglycerides exhibited no such cytotoxicity, whereas stearic acid exhibits a significant amount of toxicity.

In addition to cytotoxicity, LNPs also exhibit unpredictable genotoxicity as they are smaller in size with larger surface area and surface charges. Love et al., 2010 prepared SLNs using Witepsol and Carnauba waxes for loading siRNA against cancer. It was observed that moderate concentration of loaded SLNs showed no in vitro cyto- or genotoxicity in addition to in vivo safety profile. Löbrich et al., 2010 evaluated the genotoxicity of three SLNs formulations in hepatocarcinoma (HepG2) cells which revealed that minimal DNA damage was observed at 0.1 mg/mL of SLNs without any significantly increased DNA damage suggesting the fact that no genotoxicity at concentrations that do not reduce cell viability. Moreover, it was factualized that SLNs and NLCs are considered a safe delivery system for topical, ocular, and oral administration at lipid concentration of <1 mg/mL of total lipids. Hence, it was concluded that the cytotoxicity and genotoxicity of LNPs are dependent on the composition of the lipid matrix, the type and concentration of surfactant, and the surface electrical charge [163]. 

## 5. Conclusions

The underlying concept of LNPs as a well-tolerated carrier system is well established and documented. It was observed that the lipids employed in the preparation of LNPs such as liposomes, nanoemulsion, SLNs, NLCs, and LPH-NPs are non-toxic, biocompatible, and biodegradable with poor or no immunogenicity. The lipids have the capacity of forming nanostructure and have been investigated extensively as a nanocarrier for cancer-targeted drug delivery system. It was further concluded that the physicochemical properties of lipids provide the opportunity to optimize the drug delivery system by customizing their geometrical parameters which include particle size, morphology, entrapment efficiency, drug loading, and in vitro drug release profile. Further, the LNPs show passive targeting as well as active targeting if surface-functionalized, which enhances the therapeutic efficacy as well as targeting the cancer site. However, there exists certain reluctance regarding the efficacy and safety of LNPs which are overturned by the advent of reliable GRAS-regulated lipids, cGMP-grade manufacturing processes, and preclinical data related to their ADME status and toxicity, followed by fruitful first-in-man studies. It was further expected that the LNPs might be the first nanoparticle forming an impact on the management and treatment of cancer. Likewise, Doxil nanoparticles were observed as the first of a wave of novel LNP-mediated drug delivery systems that could deliver a transformative impact on anticancer therapeutics in the years to come.

## 6. Future Perspective

LNPs are a diverse and far-reaching type of nanoparticles that have been employed for the treatment of different types of diseases, especially cancer. However, liposomes were found to be the most successfully developed LNPs due to their flexibility and biocompatibility with biological systems. The various liposomes that shined out the lot for the treatment of cancers are Doxil^®^, Abraxane^®^, and Myocet^®^. Apart from liposomes, there are solid lipid nanoparticles (SLNs) and nanostructured lipid carriers (NLCs) that have gained a lot of interest for the treatment of malignancies from the success obtained from in vitro and in vivo studies and are considered 2nd generation LNPs. It was found that the success of such LNPs might be due to the employment of biomaterial, and environmentally safe ingredients. It was further observed that SLNs and NLCs can be used for the loading of biological drugs and imaging agents, apart from synthetic drugs. Future research is warranted in the area of targeted nanomedicine to make the concept of “Magic Bullet” a clinical reality. Interdisciplinary research is also required for the development of clinically prosperous theranostic nanoformulations.

## Figures and Tables

**Table 1:** Strategies employed for increasing the solubility of incorporated poorly water-soluble drugs by using LNPs.

Strategy	Mechanisms	Ref.
Enhanced solubilization	The lipids present in the gastrointestinal tract (GIT) increase the excretion of cholesterol and phospholipids (endogenous bile lipids), which further mediates the emulsification of the lipids present within the carrier system and solubilizes the drug.	[30]
Alteration in the biochemical barrier	Certain lipids and surfactants can decrease the intestinal secretions of the gastrointestinal wall, and prevent the metabolic activity of the enterocytes and lumen by alternating the P-glycoprotein, cytochromes, etc., thereby increasing the absorption of the drugs that are considered substrates of the stated efflux pump, and enzymes.	[25]
Alteration in the physical barrier	Some lipids and surfactants can promote intestinal absorption and membrane permeability by fluidizing the intestinal cell membrane and breaching the tight junctions.	[31]
Facilitation of lymphatic transport system	Lipids such as LCT (long-chain triglycerides) facilitate lipoprotein formation, which further facilitates their lymphatic transport. Hence, it could be stated that LNPs composed of LCT mediate lymphatic transport of poorly aqueous soluble drugs, thus bypassing the first-pass metabolism.	[25]

**Table 2:** Various types of lipid-based nanoparticles (LNPs) employed for the treatment of TNBC.

LNPs	Composition	Features	Advantages	Disadvantages	Status	Refs.
**Liposomes**	Phospholipids and cholesterol	Forms 1–20 phospholipid bilayers (vesicles) with globule size 30 nm to 3000 nm. Encapsulate hydrophilic and hydrophobic drugs.	Induce a controlled release profile. Enhances solubility of hydrophobic drugs, thereby increasing bioavailability	The structure is rigid. Controlled conditions are employed for reproducibility. Stability problems	Some are commercialized, while some are under clinical trials.	[40]
**Nanoemulsions (NEs)**	Oils, surfactants, and co-surfactants	Kinetically stable o/w dispersions. Have high surface area with small size (50–500 nm). Encapsulate both lipophilic and lipophobic drugs.	Form spontaneously. Increased reproducibility	Require high concentration of surfactant, hence can lead to toxicity. Scare choices of surfactants, as the surfactant used must be GRAS recommended.	Some are commercialized, while some are under clinical trials.	[41]
**Solid-lipid nanoparticles** **(SLNs)**	Solid lipids (fats), surfactants	Solid lipids instead of oil improve the lipidic core and provide stability and mobility to the drug within the lipidic core.	Exhibits delayed degradation of lipidic matrices allowing controlled release of the drug.	Exhibit reduced drug loading due to crystalline structure of the lipidic matrix, facilitating drug expulsion. Chances of agglomeration and polymorphic transitions.	Pre-clinical	[42]
**Nanostructured lipid carriers (NLCs)**	Solid lipids (fats), liquid lipids (fats or oils), and surfactants	NLC has a distorted structure which makes the matrix structure imperfect and creates spaces for the accommodation of active compounds.	Increased entrapment efficiency, with reduced drug leaking on storage.	Optimization required of the binary mixture, i.e., the ratio of solid and liquid lipids, otherwise, it would lead to cytotoxicity associated with the nature and concentration of lipid matrix.	Pre-clinical	[43]
**Lipid–polymer hybrid nanoparticles (LPH-NPs)**	Polymers, lipids	Hybrid vesicular structures integrate advantageous characteristics of polymers and liposomes in a single moiety.	Load efficiently one or multiple drugs with different properties.	-	Pre-clinical	[44]
**Exosomes** **(Exo)**	Cholesterol, diacylglycerol, surface proteins, heat shock proteins, lysosomal proteins, nucleic acids	Homogenous nanosized vesicles with size ranges from 30–150 nm. Formed by multivesicular bodies (MVB) after fusing with plasma membrane.	Immunocompatible	Rapid clearance from circulation after in vivo administration. No current manufacturing method.	Pre-clinical	[45,46]

**Table 3:** Summary of different LNPs employed for the treatment of TNBC.

Excipients	Results	Ref.
Liposomes		
1,2-dioleoyl-snglycero- 3-phosphocholine (DOPC) 1,2-distearoyl-sn-glycero- 3-phosphoethanolamine-N-[carboxy (polyethylene glycol)-2000] (DSPE-PEG-COOH)	- Showed an average particle size of 130 ± 30 nm with a zeta potential of −6 and −10 mV - Exhibited an enhanced internalization to TNBC cells with reduced proliferation in vitro, enhanced tumor targetability, and antitumor efficacy with reduced lung metastasis	[52]
DSPE-PEG2000	- Exhibited an enhanced cellular uptake by TNBC cells in vitro - Modified miRNA liposomes showed enhanced anticancer activity with increased internalization to TNBC cells, and increased inhibitory rates as compared to free miRNA complexes	[53]
Dioleoylphosphatidylethanolamine (DOPE)	- ILips facilitate the release of CD-47 and enhanced phagocytosis of TNBC cells and activated the responses of the T cell immune system. - ILips showed a lower IC_50_ compared to paclitaxel-liposomes and free paclitaxel - ILips showed an increased expression of CD80 (1.5-fold) as compared to free CD-47	[54]
Dipalmitoylphosphatidylcholine (DPPC) Distearoylphosphatidylcholine (DSPC) Cholesterol Distearoylphosphatidylethanolamine (DSPE)	- LipTS–GD–MAB showed increased cellular internalization as compared to doxorubicin liposomes	[55]
LecithinCholesterol	- AZD-lipo showed enhanced anti-cancer activity along with increased oral bioavailability as compared to free AZD - AZD-lipo showed decreased IC_50_ values, reduced proliferation of TNBC cells, and angiogenesis in TNBC cells as compared to free AZD	[56]
**Nanoemulsion (NEs)**		
Cod liver oil Lysophophatidylcholine (LPC), lysophophatidic acid (LPA), DSPE-PEG (2000)	- DAC/PAN LNEs decreased the cell viability of MDA-MB-231 by 55% - DAC/PAN-LNEs synergistically decreased the expression of FOXM1 mRNA and FOXM1 protein expressions by 80%	[62]
Soya lecithin Kolliphor^®^ HS15	- NanoPue reduced the expression of TAFs and enhanced ITLs of cytotoxic T cells by 6-fold and 2-fold respectively as compared to control	[63]
Soybean phospholipids Cholesterol	- NE reduced the stabilization of HIF-1α by effectively scavenging ROS. - NE limited angiogenesis and NLRP3 inflammasomes and IL-1β	[64]
Miglyol 812 Phosphatidylcholine	- NEs decreased tumor growth and cell proliferation in vitro and in vivo. - ET-NEs showed a dose-dependent IC_50_ which was found to be 6.9 μg/mL at 13.2 μM after 24 h of incubation, whereas the free ET showed a higher IC_50_ which is 13.9 μg/mL at 26.5 μM	[65]
**Solid lipid nanoparticles (SLNs)**		
GMS (Glyceryl monostearate) Tween 80	- BMN 673-SLNs induced significant toxicity in TNBC cells	[75]
Palmitic acid Pluronic F-68 Soy lecithin	- DADS-RAGE-SLNs significantly increased the cytotoxicity, apoptosis and cellular internalization as compared to DADS	[76]
GMS SA (Stearic acid) Compritol ATO 888 Tween 80 as a surfactant	- DTX-ALA-SLNs showed increased cytotoxicity to 4T1 cells as compared to DTX-SLNs, ALA-SLNs, and free drugs - Also, DTX-ALA SLNs showed increased apoptosis of 32% as compared to free DTX which is only 11%	[77]
Stearyl amine Tween 80, Pluronic F-68	- Niclo-SLNs showed increased cytotoxicity and enhanced cellular internalization at the G0/G1 phase of the cell cycle as compared to free Niclo	[78]
	- PBA-Niclo-SLNs showed increased cytotoxicity, inhibition of cell proliferation at G0/G1 cell cycle and apoptosis as compared to Niclo-SLNs and free Niclo respectively. - PBA-Niclo-SLNs significantly inhibited STAT3, TNBC stem cell populations, and EMT markers	[79]
**Nanostructured lipid carriers (NLCs)**		
Compritol ATO 888 Medium chain triglycerides (MCT) Tween 80 Soya lecithin	- PTX-NLCs showed increased *in-vitro* cell cytotoxicity and anti-clonogenic activity against MDA-MB-231 cells as compared to free PTX - PTX-NLC exhibited 1.5 and 1.7-fold increased tumor site accumulation after 30 and 120 min respectively in tumor-bearing mice, as compared to free PTX	[81]
Precirol ATO 5 Maisine 35-1 Cremophor RH40	- FA-PTX-Ce6-NLC showed enhanced MDA-MB-231 cellular uptake as compared to free PTX - NLC system also showed enhanced drug-loading without side effects as compared to free PTX	[82]
Compritol 888 ATO Docosahexaenoic acid (DHA) Tween 80	- NLCs showed a controlled release profile with an increased release in acidic media - NLCs exhibited decreased mortality in mice, reduced metastasis to lungs, prevented drug-induced toxicity to vital organs	[83]
GMS Caproyl 90 Labrasol	- RVT-NLC showed decreased cell-viability and increased therapeutic efficacy as compared to free RVT - Further, RVT-NLCs loaded microneedle showed increased skin permeation, improved cellular internalization, increased pharmacokinetic attributes and prevented metastasis as compared to free RVT	[84]
GMS Caproyl 90 Poloxamer 188	- LTN-CS-NLC exhibited a slow-release profile of LTN during a 24 h study with increased mucoadhesion, improved gastrointestinal stability, and intestinal permeation as compared to free LTN. - Moreover, LTN-CS-NLC showed decreased MDA-MB-231 cell viability as compared to free LTN after 48 h treatment	[85]
**Lipid–** **Polymer hybrid nanoparticles (LPH-NPs)**		
HPESO (hydrolyzed polymer of epoxidized soyabean oil) Myristic acid	- RGD-DMPLN increased cytotoxicity, cellular accumulation, restricted lung metastasis (31-fold), decreased toxicity to the liver and heart, and improved median survival time (57%)	[87]
Poly lactide glycolic acid (PLGA) Polyethylene glycol (PEG) Dioleoylphosphatidic acid (DOPA)	- LPH-NPs decreased the cell viability by approximately 80% as compared to free paclitaxel at the same dose of 0.67 μg/mL - Moreover, LPH-NPs showed enhanced intracellular activity as compared to free paclitaxel	[88]
Gelucire 48/16, Phospholipid 90NG Phospholipid S100	- LPH-NPs exhibited rapid cellular internalization within 2 h, showed 10-fold increased bioavailability, ~21–25% less tumor cell growth, and 5–6 times increased MRT as compared to free drugs	[89]
PLGA, DSPE-PEG Lecithin	- CuB-NPs showed decreased cell viability, increased apoptosis as compared to free CuB	[90]
**Exosomes (Exo)**		
Mesenchymal stem cells (MSCs), Surface proteins: tetraspanins (CD63, CD9, CD81), heat shock proteins (Hsc70), lysosomal proteins (Lamp2b), and fusion proteins (flotillin, annexin).	- MSCs-Exo efficiently delivered anti-miR-142-3p to TNBC cells - Increased the transcription of the regulatory target genes. - MSCs-Exo exhibited enhanced penetration to cancer cells.	[101]
Human monocyte-derived macrophage cells Surface proteins (Exosomal marker proteins): CD81 and CD63.	- A15-Exo co-loaded with Dox and Cho-miR159 exhibited synergistic therapeutic activity. - miR159 and Dox delivery effectively silenced the TCF-7 gene and showed enhanced anticancer effects, without any adverse effects	[102]
Human fetal lung fibroblast Surface proteins (Exosomal markers): TSG101 and CD81	- Erastin@FA-exo showed increased cellular uptake compared to free erastin. - Moreover, showed better inhibitory effect on the proliferation and migration of TNBC cells. - Erastin@FA-exo showed enhanced ferroptosis with intracellular depletion of glutathione and ROS production.	[103]
Macrophage Surface proteins	- Engineered exosome coated nanoparticles exhibited increased cellular uptake and enhanced antitumor efficacy compared to free Dox and Dox loaded polymeric nanoparticles. - Moreover, engineered exosome coated nanocarriers demonstrated remarkable tumor-targetability that further led to significant inhibition of tumor growth and tumor apoptosis.	[104]

## Data Availability

Not applicable.
